# Bis[μ-bis­(diphenyl­phosphino)methane-κ^2^ *P*:*P*′]bis­[(2,2′-bipyridine-κ^2^ *N*,*N*′)copper(I)] bis­(tetra­fluoro­borate)

**DOI:** 10.1107/S1600536809038252

**Published:** 2009-09-26

**Authors:** Shouwen Jin, Daqi Wang

**Affiliations:** a Faculty of Science, Zhejiang Forestry University, Lin’An 311300, People’s Republic of China; b Department of Chemistry, Liaocheng University, Liaocheng 52059, People’s Republic of China

## Abstract

The centrosymmetric title compound, [Cu_2_(C_10_H_8_N_2_)_2_(C_25_H_22_P_2_)_2_](BF_4_)_2_, consists of discrete dinuclear cations and tetra­fluoro­borate anions. The two Cu^I^ centers are bridged by the phosphine ligands to form an eight-membered ring. The Cu^I^ center exhibits a tetra­hedral coordination as it is chelated by the *N*-heterocycle.

## Related literature

For general background to binuclear metal complexes containing bis(diphenylphosphino)methane, see: Stockland *et al.* (2001 ▶); Jin *et al.* (2008 ▶). For their photochemical and photophysical properties, see: Armaroli (2001 ▶); Yam *et al.* (1997 ▶). For related structures, see: Diez *et al.* (1987 ▶); Ho & Bau (1983 ▶); Kuang *et al.* (2002 ▶). For the synthesis, see: Jia *et al.* (2005 ▶).
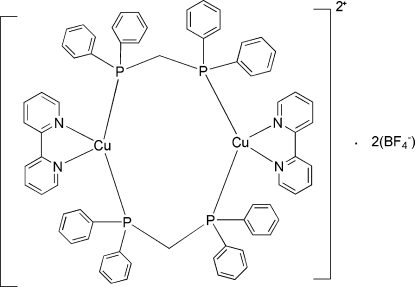


## Experimental

### 

#### Crystal data

[Cu_2_(C_10_H_8_N_2_)_2_(C_25_H_22_P_2_)_2_](BF_4_)_2_
*M* *_r_* = 1381.80
Triclinic, 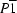

*a* = 11.5601 (11) Å
*b* = 12.1936 (13) Å
*c* = 13.1022 (18) Å
α = 64.603 (1)°
β = 75.781 (2)°
γ = 75.120 (2)°
*V* = 1592.9 (3) Å^3^
*Z* = 1
Mo *K*α radiation
μ = 0.84 mm^−1^
*T* = 298 K
0.35 × 0.29 × 0.17 mm

#### Data collection

Bruker SMART diffractometer
Absorption correction: multi-scan (*SADABS*; Sheldrick, 1996 ▶) *T*_min_ = 0.758, *T*_max_ = 0.871
8214 measured reflections
5497 independent reflections
3264 reflections with *I* > 2σ(*I*)
*R*_int_ = 0.052

#### Refinement

*R*[*F* ^2^ > 2σ(*F* ^2^)] = 0.078
*wR*(*F* ^2^) = 0.216
*S* = 1.12
5497 reflections
434 parameters
H-atom parameters constrained
Δρ_max_ = 1.44 e Å^−3^
Δρ_min_ = −0.80 e Å^−3^

### 

Data collection: *SMART* (Bruker, 1997 ▶); cell refinement: *SAINT* (Bruker, 1997 ▶); data reduction: *SAINT*; program(s) used to solve structure: *SHELXS97* (Sheldrick, 2008 ▶); program(s) used to refine structure: *SHELXL97* (Sheldrick, 2008 ▶); molecular graphics: *SHELXTL* (Sheldrick, 2008 ▶); software used to prepare material for publication: *SHELXTL*.

## Supplementary Material

Crystal structure: contains datablocks global, I. DOI: 10.1107/S1600536809038252/ng2640sup1.cif

Structure factors: contains datablocks I. DOI: 10.1107/S1600536809038252/ng2640Isup2.hkl

Additional supplementary materials:  crystallographic information; 3D view; checkCIF report

## Figures and Tables

**Fig. 1.**
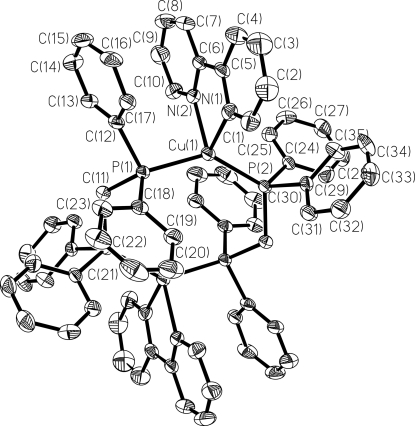
The structure of the dimeric cation, showing the atom-numbering scheme. Displacement ellipsoids are drawn at the 30% probability level.

## supplementary crystallographic information

### Comment

The bis(diphenylphosphino)methane (dppm) is an important bridging ligand. During the past decade transition metal chemistry of dppm have been widely studied. A large number of binuclear metal complexes containing dppm are known with a variety of photophysics and stereochemistry (Diez *et al.*, 1987; Stockland *et al.*, 2001; Yam *et al.*, 1997).

Copper(I) complexes, which are inexpensive, abundant, and as strongly emissive as d^10^ Au(I) complexes, have received increased attention (Armaroli, 2001). As we are interested in research of Cu^I^ complexes containing N-heterocyclic compound and phosphine compounds (Jin *et al.*, 2008). To obtain further insight into this very interesting field of research, we therefore decided to initiate an investigation on substituting the dppe(bis(diphenylphosphino)ethane) with dppm. In this work, we report on the reaction of [Cu_2_(dppm)_2_(CH_3_CN)_2_](BF_4_)_2_ with 2,2'-bipyridine, as well as the structure of [Cu_2_(dppm)_2_(*L*)_2_]_2_(BF_4_)_2_.

The complex was prepared by reacting equal mol of [Cu_2_(dppm)_2_(CH_3_CN)_2_](BF_4_)_2_, and 2,2'-bipyridine (*L*) in dry ethanol solution. This procedure, frequently used for the preparation of copper(I) complexes containing both N-heterocyclic compounds and triphenylphosphine ligands, gave dinuclear complex of the formula [Cu_2_(PPh_3_)_4_L](BF_4_)_2_ (Jia *et al.*, 2005). The compound is an ionic compound which consists of dimeric [Cu_2_(µ-dppm)_2_(*L*)_2_]^2+^ cations, and of tetrafluoroborate anions. The asymmetric unit of dimeric [Cu_2_(µ-dppm)_2_(*L*)_2_]^2+^ cations contains a half of the cations including a Cu^I^ atom, one 2,2'-bipyridine, and one bis(diphenylphosphino)methane of which the phosphorus donor atoms are in *cis*-bound position. The structure of the cation is depicted in Fig. 1 together with the atomic numbering scheme. Two copper atoms are doubly bridged by two dppm ligands to form an eight-membered Cu_2_P_4_C_2_ ring, which displays chair conformation. The slightly distorted tetrahedral coordination around the copper atom is completed by two nitrogen atoms from chelate 2,2'-bipyridine ligands. Copper atoms doubly bridged by two dppm ligands have been found also in [Cu_2_(µ-dppm)_2_(MeCN)_4_][ClO_4_]_2_ (Diez *et al.*, 1987), although the Cu—Cu separation of 4.599 Å in this investigation is longer than that found in complex [Cu_2_(µ-dppm)_2_(MeCN)_4_][ClO_4_]_2_ (3.757 (3) Å) (Diez *et al.*, 1987), which may be due to the crowdiness of the coordinated 2,2'-bipyridine. The corresponding Cu—N bond lengths are 2.094 (6), and 2.106 (5) Å respectively being much similar to Cu—N (2.104 (3) Å) bond distance of [Cu(dmp)(DPEphos)]BF_4_ (Kuang *et al.*, 2002). Also the two Cu—P bonds have a significant difference, one Cu—P bond (Cu(1)—P(2) 2.238 (2) Å) in the title compound is shorter than those in [Cu_2_(µ-dppm)_2_(MeCN)_4_][ClO_4_]_2_ (2.270 (3) and 2.283 (3) Å), another Cu—P (Cu(1)—P(1) 2.283 (2) Å) bond is almost the same with the value in [Cu_2_(µ-dppm)_2_(MeCN)_4_][ClO_4_]_2_ (Diez *et al.*, 1987). The irregularities in the tetrahedral coordination geometry about the copper(I) center are best reflected in the values of the bond angles, since only three of them are close to the ideal tetrahedral value. For the d*^l^*^0^ complexes, the P—M—P units are distinctly nonlinear and *M*_2_P_4_ skeletal units are not coplanar (Ho & Bau, 1983), our compound conforms with this case also. As expected, the largest angle P(2)—Cu(1)—P(1) (136.81 (6) °) which is larger than the corresponding value in [Cu_2_(µ-dppm)_2_(MeCN)_4_][ClO_4_]_2_ (Diez *et al.*, 1987)arises between the two most bulky ligands.

### Experimental

The CHN elemental analyses were performed on a Perkin-Elmer elemental analyzer.

To a solution of 2,2'-bipyridine (0.032 g, 0.2 mmol) in 10 ml of ethanol was added [Cu_2_(dppm)_2_(CH_3_CN)_2_](BF_4_)_2_ (0.180 g, 0.2 mmol). The mixture was stirred at room temperature overnight to afford a yellow solid, which was collected by filtration, washed with ethanol and ether, Yield: 0.148 g, 53.5%. Anal. Calcd. for C_70_H_60_B_2_Cu_2_F_8_N_4_P_4_: C, 60.83%, H, 4.34%, N, 4.06%. Found: C, 60.81%, H, 4.28%, N, 4.13%. Suitable crystals were grown by slow diffusion of diethyl ether to its DMF solution.

### Refinement

All H atoms were placed in geometrically idealized positions and constrained to ride on their parent atoms, with C—H = 0.93–0.97 Å, and *U*_iso_(H) = 1.2Ueq(C).

The largest peak/hole in the difference Fourier map are 1.435 and -0.802 respectively.

### Crystal data

**Table:**

[Cu_2_(C_10_H_8_N_2_)_2_(C_25_H_22_P_2_)_2_](BF_4_)_2_	*Z* = 1
*M**_r_* = 1381.80	*F*(000) = 708
Triclinic, *P*1	*D*_x_ = 1.440 Mg m^−^^3^
*a* = 11.5601 (11) Å	Mo *K*α radiation, λ = 0.71073 Å
*b* = 12.1936 (13) Å	Cell parameters from 1867 reflections
*c* = 13.1022 (18) Å	θ = 2.3–22.2°
α = 64.603 (1)°	µ = 0.84 mm^−^^1^
β = 75.781 (2)°	*T* = 298 K
γ = 75.120 (2)°	Prism, yellow
*V* = 1592.9 (3) Å^3^	0.35 × 0.29 × 0.17 mm

### Data collection

**Table:**

Bruker SMART diffractometer	5497 independent reflections
Radiation source: fine-focus sealed tube	3264 reflections with *I* > 2σ(*I*)
graphite	*R*_int_ = 0.052
φ and ω scans	θ_max_ = 25.0°, θ_min_ = 1.7°
Absorption correction: multi-scan (*SADABS*; Sheldrick, 1996)	*h* = −13→11
*T*_min_ = 0.758, *T*_max_ = 0.871	*k* = −14→14
8214 measured reflections	*l* = −15→12

### Refinement

**Table:**

Refinement on *F*^2^	Primary atom site location: structure-invariant direct methods
Least-squares matrix: full	Secondary atom site location: difference Fourier map
*R*[*F*^2^ > 2σ(*F*^2^)] = 0.078	Hydrogen site location: inferred from neighbouring sites
*wR*(*F*^2^) = 0.216	H-atom parameters constrained
*S* = 1.12	*w* = 1/[σ^2^(*F*_o_^2^) + (0.0959*P*)^2^] where *P* = (*F*_o_^2^ + 2*F*_c_^2^)/3
5497 reflections	(Δ/σ)_max_ = 0.001
434 parameters	Δρ_max_ = 1.44 e Å^−^^3^
0 restraints	Δρ_min_ = −0.80 e Å^−^^3^

### Special details

**Table:**

Geometry. All e.s.d.'s (except the e.s.d. in the dihedral angle between two l.s. planes) are estimated using the full covariance matrix. The cell e.s.d.'s are taken into account individually in the estimation of e.s.d.'s in distances, angles and torsion angles; correlations between e.s.d.'s in cell parameters are only used when they are defined by crystal symmetry. An approximate (isotropic) treatment of cell e.s.d.'s is used for estimating e.s.d.'s involving l.s. planes.
Refinement. Refinement of *F*^2^ against ALL reflections. The weighted *R*-factor *wR* and goodness of fit *S* are based on *F*^2^, conventional *R*-factors *R* are based on *F*, with *F* set to zero for negative *F*^2^. The threshold expression of *F*^2^ > σ(*F*^2^) is used only for calculating *R*-factors(gt) *etc*. and is not relevant to the choice of reflections for refinement. *R*-factors based on *F*^2^ are statistically about twice as large as those based on *F*, and *R*- factors based on ALL data will be even larger.

### Fractional atomic coordinates and isotropic or equivalent isotropic displacement parameters (Å^2^)

**Table:**

	*x*	*y*	*z*	*U*_iso_*/*U*_eq_	Occ. (<1)
Cu1	0.50912 (7)	0.43927 (7)	0.35411 (6)	0.0397 (3)	
B1	0.7558 (10)	0.8853 (10)	0.1027 (8)	0.067 (3)	
F1	0.7993 (5)	0.9609 (5)	0.1329 (4)	0.0951 (16)	
F2	0.645 (5)	0.941 (8)	0.066 (9)	0.079 (10)	0.6 (3)
F3	0.839 (5)	0.868 (9)	0.011 (6)	0.087 (8)	0.6 (3)
F4	0.749 (11)	0.773 (6)	0.193 (5)	0.087 (14)	0.6 (3)
F2'	0.666 (12)	0.955 (10)	0.035 (9)	0.078 (12)	0.4 (3)
F3'	0.701 (16)	0.801 (12)	0.203 (4)	0.090 (16)	0.4 (3)
F4'	0.845 (5)	0.822 (19)	0.047 (16)	0.09 (2)	0.4 (3)
N1	0.5285 (5)	0.2987 (5)	0.2966 (5)	0.0493 (14)	
N2	0.5743 (5)	0.5269 (5)	0.1790 (4)	0.0447 (13)	
P1	0.68607 (14)	0.38802 (15)	0.42487 (13)	0.0363 (4)	
P2	0.31095 (14)	0.47660 (14)	0.41841 (13)	0.0347 (4)	
C1	0.5006 (7)	0.1871 (7)	0.3560 (7)	0.060 (2)	
H1	0.4710	0.1653	0.4336	0.072*	
C2	0.5126 (8)	0.1007 (8)	0.3098 (9)	0.079 (3)	
H2	0.4918	0.0234	0.3549	0.094*	
C3	0.5562 (9)	0.1334 (9)	0.1957 (10)	0.093 (3)	
H3	0.5648	0.0785	0.1612	0.111*	
C4	0.5874 (8)	0.2486 (9)	0.1322 (8)	0.084 (3)	
H4	0.6173	0.2719	0.0545	0.101*	
C5	0.5737 (6)	0.3292 (7)	0.1850 (6)	0.0530 (18)	
C6	0.6058 (6)	0.4537 (7)	0.1216 (6)	0.0478 (17)	
C7	0.6681 (8)	0.4930 (9)	0.0094 (6)	0.072 (2)	
H7	0.6900	0.4411	−0.0300	0.086*	
C8	0.6957 (8)	0.6078 (9)	−0.0406 (7)	0.083 (3)	
H8	0.7371	0.6351	−0.1151	0.100*	
C9	0.6636 (8)	0.6832 (9)	0.0169 (7)	0.079 (3)	
H9	0.6821	0.7623	−0.0170	0.095*	
C10	0.6031 (6)	0.6396 (7)	0.1266 (6)	0.0568 (19)	
H10	0.5811	0.6910	0.1665	0.068*	
C11	0.7495 (6)	0.4990 (5)	0.4462 (5)	0.0388 (15)	
H11A	0.7360	0.5779	0.3828	0.047*	
H11B	0.8365	0.4719	0.4426	0.047*	
C12	0.8068 (6)	0.3515 (6)	0.3173 (5)	0.0410 (15)	
C13	0.8772 (6)	0.4376 (7)	0.2381 (5)	0.0519 (18)	
H13	0.8689	0.5132	0.2430	0.062*	
C14	0.9579 (7)	0.4115 (9)	0.1541 (6)	0.073 (2)	
H14	1.0053	0.4695	0.1024	0.087*	
C15	0.9717 (7)	0.3019 (9)	0.1431 (6)	0.070 (2)	
H15	1.0289	0.2853	0.0855	0.084*	
C16	0.8997 (7)	0.2156 (8)	0.2183 (7)	0.068 (2)	
H16	0.9049	0.1427	0.2094	0.081*	
C17	0.8198 (6)	0.2406 (7)	0.3070 (6)	0.0503 (17)	
H17	0.7743	0.1817	0.3604	0.060*	
C18	0.7024 (6)	0.2477 (6)	0.5546 (5)	0.0434 (16)	
C19	0.6008 (7)	0.2131 (7)	0.6317 (6)	0.0568 (19)	
H19	0.5247	0.2569	0.6144	0.068*	
C20	0.6108 (11)	0.1126 (9)	0.7358 (7)	0.084 (3)	
H20	0.5419	0.0916	0.7889	0.101*	
C21	0.7213 (12)	0.0459 (8)	0.7592 (8)	0.086 (3)	
H21	0.7279	−0.0225	0.8276	0.103*	
C22	0.8256 (9)	0.0790 (7)	0.6813 (7)	0.074 (2)	
H22	0.9015	0.0329	0.6972	0.088*	
C23	0.8146 (7)	0.1794 (6)	0.5820 (6)	0.0549 (18)	
H23	0.8842	0.2030	0.5310	0.066*	
C24	0.2196 (5)	0.6013 (6)	0.3136 (5)	0.0378 (14)	
C25	0.2751 (6)	0.6778 (6)	0.2144 (5)	0.0461 (16)	
H25	0.3593	0.6671	0.2001	0.055*	
C26	0.2088 (7)	0.7724 (7)	0.1330 (6)	0.059 (2)	
H26	0.2483	0.8256	0.0661	0.071*	
C27	0.0844 (7)	0.7861 (7)	0.1526 (7)	0.066 (2)	
H27	0.0390	0.8474	0.0984	0.079*	
C28	0.0279 (7)	0.7079 (7)	0.2535 (7)	0.066 (2)	
H28	−0.0562	0.7167	0.2667	0.080*	
C29	0.0928 (6)	0.6180 (7)	0.3343 (6)	0.0580 (19)	
H29	0.0530	0.5678	0.4030	0.070*	
C30	0.2459 (5)	0.3410 (6)	0.4496 (5)	0.0387 (15)	
C31	0.2526 (6)	0.2395 (6)	0.5525 (5)	0.0486 (17)	
H31	0.2842	0.2428	0.6097	0.058*	
C32	0.2132 (7)	0.1342 (7)	0.5712 (6)	0.060 (2)	
H32	0.2182	0.0675	0.6410	0.072*	
C33	0.1666 (7)	0.1261 (7)	0.4878 (7)	0.066 (2)	
H33	0.1394	0.0552	0.5006	0.079*	
C34	0.1615 (7)	0.2254 (7)	0.3860 (7)	0.066 (2)	
H34	0.1317	0.2208	0.3284	0.079*	
C35	0.1991 (7)	0.3318 (7)	0.3662 (6)	0.0581 (19)	
H35	0.1932	0.3983	0.2964	0.070*	

### Atomic displacement parameters (Å^2^)

**Table:**

	*U*^11^	*U*^22^	*U*^33^	*U*^12^	*U*^13^	*U*^23^
Cu1	0.0353 (5)	0.0503 (5)	0.0409 (5)	−0.0031 (4)	−0.0079 (3)	−0.0260 (4)
B1	0.068 (7)	0.088 (8)	0.054 (6)	0.001 (6)	−0.027 (5)	−0.035 (6)
F1	0.088 (4)	0.112 (4)	0.103 (4)	−0.009 (3)	−0.028 (3)	−0.056 (3)
F2	0.055 (13)	0.12 (2)	0.06 (2)	0.003 (10)	−0.013 (14)	−0.04 (2)
F3	0.065 (11)	0.12 (2)	0.068 (16)	0.019 (12)	−0.018 (9)	−0.044 (19)
F4	0.09 (3)	0.093 (17)	0.078 (11)	−0.021 (16)	−0.044 (16)	−0.014 (9)
F2'	0.06 (3)	0.12 (2)	0.04 (2)	0.000 (19)	−0.017 (18)	−0.03 (2)
F3'	0.09 (4)	0.12 (2)	0.062 (12)	−0.01 (3)	−0.031 (17)	−0.027 (14)
F4'	0.067 (11)	0.13 (5)	0.09 (4)	0.03 (2)	−0.038 (18)	−0.07 (4)
N1	0.039 (3)	0.057 (4)	0.058 (4)	−0.006 (3)	−0.004 (3)	−0.031 (3)
N2	0.034 (3)	0.060 (4)	0.045 (3)	−0.007 (3)	−0.004 (2)	−0.027 (3)
P1	0.0317 (9)	0.0466 (10)	0.0361 (9)	−0.0026 (7)	−0.0063 (6)	−0.0230 (8)
P2	0.0334 (9)	0.0424 (9)	0.0345 (8)	−0.0041 (7)	−0.0087 (6)	−0.0201 (8)
C1	0.055 (5)	0.054 (5)	0.074 (5)	−0.005 (4)	−0.004 (4)	−0.033 (4)
C2	0.076 (6)	0.058 (5)	0.120 (8)	0.001 (4)	−0.015 (5)	−0.059 (6)
C3	0.100 (8)	0.087 (7)	0.121 (9)	−0.003 (6)	−0.010 (6)	−0.080 (7)
C4	0.083 (7)	0.115 (8)	0.091 (7)	−0.018 (6)	−0.001 (5)	−0.081 (7)
C5	0.041 (4)	0.080 (5)	0.063 (5)	−0.002 (4)	−0.012 (3)	−0.054 (4)
C6	0.035 (4)	0.073 (5)	0.049 (4)	−0.005 (3)	−0.008 (3)	−0.038 (4)
C7	0.076 (6)	0.110 (7)	0.050 (5)	−0.026 (5)	−0.001 (4)	−0.048 (5)
C8	0.099 (7)	0.115 (8)	0.042 (5)	−0.046 (6)	0.010 (4)	−0.032 (5)
C9	0.090 (7)	0.091 (6)	0.051 (5)	−0.043 (5)	−0.011 (4)	−0.008 (5)
C10	0.053 (5)	0.063 (5)	0.056 (5)	−0.010 (4)	−0.002 (3)	−0.027 (4)
C11	0.040 (4)	0.045 (4)	0.037 (3)	−0.009 (3)	−0.005 (3)	−0.021 (3)
C12	0.038 (4)	0.050 (4)	0.037 (3)	0.011 (3)	−0.015 (3)	−0.025 (3)
C13	0.051 (4)	0.070 (5)	0.040 (4)	−0.007 (4)	−0.005 (3)	−0.029 (4)
C14	0.065 (6)	0.102 (7)	0.051 (5)	−0.015 (5)	0.001 (4)	−0.034 (5)
C15	0.061 (5)	0.101 (7)	0.047 (5)	0.009 (5)	−0.003 (4)	−0.044 (5)
C16	0.068 (5)	0.086 (6)	0.065 (5)	0.027 (5)	−0.022 (4)	−0.060 (5)
C17	0.043 (4)	0.064 (5)	0.050 (4)	0.000 (3)	−0.011 (3)	−0.032 (4)
C18	0.053 (4)	0.046 (4)	0.041 (4)	−0.010 (3)	−0.008 (3)	−0.025 (3)
C19	0.066 (5)	0.066 (5)	0.047 (4)	−0.023 (4)	−0.006 (4)	−0.025 (4)
C20	0.132 (9)	0.085 (7)	0.050 (5)	−0.069 (7)	0.001 (5)	−0.021 (5)
C21	0.146 (10)	0.053 (5)	0.060 (6)	−0.025 (6)	−0.042 (7)	−0.005 (5)
C22	0.101 (7)	0.057 (5)	0.066 (5)	0.013 (5)	−0.036 (5)	−0.030 (5)
C23	0.067 (5)	0.052 (4)	0.049 (4)	0.000 (4)	−0.019 (4)	−0.022 (4)
C24	0.036 (4)	0.045 (4)	0.042 (4)	−0.004 (3)	−0.012 (3)	−0.023 (3)
C25	0.041 (4)	0.049 (4)	0.045 (4)	0.004 (3)	−0.011 (3)	−0.020 (3)
C26	0.076 (6)	0.053 (4)	0.037 (4)	−0.003 (4)	−0.002 (4)	−0.015 (4)
C27	0.059 (5)	0.065 (5)	0.073 (5)	0.011 (4)	−0.034 (4)	−0.024 (5)
C28	0.046 (5)	0.073 (5)	0.069 (5)	−0.002 (4)	−0.027 (4)	−0.011 (5)
C29	0.045 (4)	0.070 (5)	0.052 (4)	−0.008 (4)	−0.009 (3)	−0.017 (4)
C30	0.031 (3)	0.045 (4)	0.043 (4)	−0.005 (3)	−0.009 (3)	−0.019 (3)
C31	0.056 (4)	0.054 (4)	0.042 (4)	−0.011 (3)	−0.012 (3)	−0.022 (4)
C32	0.070 (5)	0.053 (5)	0.055 (5)	−0.018 (4)	−0.009 (4)	−0.015 (4)
C33	0.070 (6)	0.049 (5)	0.088 (6)	−0.024 (4)	−0.012 (5)	−0.029 (5)
C34	0.077 (6)	0.066 (5)	0.081 (6)	−0.017 (4)	−0.033 (4)	−0.038 (5)
C35	0.072 (5)	0.063 (5)	0.049 (4)	−0.010 (4)	−0.022 (4)	−0.026 (4)

### Geometric parameters (Å, °)

**Table:**

Cu1—N1	2.094 (5)	C13—C14	1.351 (9)
Cu1—N2	2.106 (5)	C13—H13	0.9300
Cu1—P2	2.2384 (17)	C14—C15	1.371 (11)
Cu1—P1	2.2830 (18)	C14—H14	0.9300
B1—F4'	1.38 (5)	C15—C16	1.388 (11)
B1—F4	1.38 (3)	C15—H15	0.9300
B1—F2	1.38 (6)	C16—C17	1.389 (9)
B1—F1	1.381 (11)	C16—H16	0.9300
B1—F2'	1.39 (10)	C17—H17	0.9300
B1—F3'	1.40 (6)	C18—C19	1.373 (9)
B1—F3	1.40 (3)	C18—C23	1.386 (9)
N1—C1	1.325 (8)	C19—C20	1.395 (11)
N1—C5	1.344 (8)	C19—H19	0.9300
N2—C6	1.329 (8)	C20—C21	1.354 (13)
N2—C10	1.336 (8)	C20—H20	0.9300
P1—C18	1.830 (7)	C21—C22	1.396 (13)
P1—C11	1.840 (6)	C21—H21	0.9300
P1—C12	1.840 (6)	C22—C23	1.358 (10)
P2—C30	1.837 (6)	C22—H22	0.9300
P2—C24	1.847 (6)	C23—H23	0.9300
P2—C11^i^	1.860 (6)	C24—C25	1.352 (8)
C1—C2	1.387 (10)	C24—C29	1.402 (9)
C1—H1	0.9300	C25—C26	1.396 (9)
C2—C3	1.368 (13)	C25—H25	0.9300
C2—H2	0.9300	C26—C27	1.377 (10)
C3—C4	1.380 (12)	C26—H26	0.9300
C3—H3	0.9300	C27—C28	1.377 (10)
C4—C5	1.385 (10)	C27—H27	0.9300
C4—H4	0.9300	C28—C29	1.364 (10)
C5—C6	1.479 (9)	C28—H28	0.9300
C6—C7	1.401 (10)	C29—H29	0.9300
C7—C8	1.353 (11)	C30—C35	1.387 (9)
C7—H7	0.9300	C30—C31	1.388 (8)
C8—C9	1.354 (11)	C31—C32	1.375 (9)
C8—H8	0.9300	C31—H31	0.9300
C9—C10	1.370 (10)	C32—C33	1.379 (10)
C9—H9	0.9300	C32—H32	0.9300
C10—H10	0.9300	C33—C34	1.366 (10)
C11—P2^i^	1.860 (6)	C33—H33	0.9300
C11—H11A	0.9700	C34—C35	1.374 (9)
C11—H11B	0.9700	C34—H34	0.9300
C12—C17	1.382 (9)	C35—H35	0.9300
C12—C13	1.390 (9)		
			
N1—Cu1—N2	78.6 (2)	P2^i^—C11—H11A	108.1
N1—Cu1—P2	104.72 (15)	P1—C11—H11B	108.1
N2—Cu1—P2	119.60 (15)	P2^i^—C11—H11B	108.1
N1—Cu1—P1	101.00 (16)	H11A—C11—H11B	107.3
N2—Cu1—P1	99.03 (15)	C17—C12—C13	118.7 (6)
P2—Cu1—P1	136.81 (6)	C17—C12—P1	119.3 (5)
F4'—B1—F4	86 (5)	C13—C12—P1	121.6 (5)
F4'—B1—F2	122 (3)	C14—C13—C12	120.0 (7)
F4—B1—F2	112 (2)	C14—C13—H13	120.0
F4'—B1—F1	113 (4)	C12—C13—H13	120.0
F4—B1—F1	109.9 (18)	C13—C14—C15	121.7 (8)
F2—B1—F1	111 (3)	C13—C14—H14	119.1
F4'—B1—F2'	111 (4)	C15—C14—H14	119.1
F4—B1—F2'	126 (4)	C14—C15—C16	119.7 (7)
F2—B1—F2'	17 (3)	C14—C15—H15	120.2
F1—B1—F2'	109 (5)	C16—C15—H15	120.2
F4'—B1—F3'	109 (4)	C15—C16—C17	118.6 (7)
F4—B1—F3'	24 (3)	C15—C16—H16	120.7
F2—B1—F3'	91 (4)	C17—C16—H16	120.7
F1—B1—F3'	107 (2)	C12—C17—C16	121.2 (7)
F2'—B1—F3'	107 (4)	C12—C17—H17	119.4
F4'—B1—F3	23 (6)	C16—C17—H17	119.4
F4—B1—F3	110 (2)	C19—C18—C23	118.4 (7)
F2—B1—F3	108.1 (18)	C19—C18—P1	119.1 (6)
F1—B1—F3	106.7 (18)	C23—C18—P1	122.4 (5)
F2'—B1—F3	93 (3)	C18—C19—C20	120.6 (8)
F3'—B1—F3	131 (3)	C18—C19—H19	119.7
C1—N1—C5	117.9 (6)	C20—C19—H19	119.7
C1—N1—Cu1	128.0 (5)	C21—C20—C19	119.7 (8)
C5—N1—Cu1	114.1 (5)	C21—C20—H20	120.1
C6—N2—C10	118.2 (6)	C19—C20—H20	120.1
C6—N2—Cu1	114.5 (4)	C20—C21—C22	120.5 (8)
C10—N2—Cu1	126.4 (5)	C20—C21—H21	119.8
C18—P1—C11	104.8 (3)	C22—C21—H21	119.8
C18—P1—C12	103.4 (3)	C23—C22—C21	119.1 (8)
C11—P1—C12	100.2 (3)	C23—C22—H22	120.5
C18—P1—Cu1	116.5 (2)	C21—C22—H22	120.5
C11—P1—Cu1	122.8 (2)	C22—C23—C18	121.7 (7)
C12—P1—Cu1	106.3 (2)	C22—C23—H23	119.2
C30—P2—C24	102.6 (3)	C18—C23—H23	119.2
C30—P2—C11^i^	100.2 (3)	C25—C24—C29	118.9 (6)
C24—P2—C11^i^	104.2 (3)	C25—C24—P2	120.0 (5)
C30—P2—Cu1	108.4 (2)	C29—C24—P2	121.1 (5)
C24—P2—Cu1	115.4 (2)	C24—C25—C26	121.4 (7)
C11^i^—P2—Cu1	123.3 (2)	C24—C25—H25	119.3
N1—C1—C2	124.1 (8)	C26—C25—H25	119.3
N1—C1—H1	117.9	C27—C26—C25	119.3 (7)
C2—C1—H1	117.9	C27—C26—H26	120.3
C3—C2—C1	117.6 (8)	C25—C26—H26	120.3
C3—C2—H2	121.2	C26—C27—C28	119.3 (7)
C1—C2—H2	121.2	C26—C27—H27	120.4
C2—C3—C4	119.3 (8)	C28—C27—H27	120.4
C2—C3—H3	120.3	C29—C28—C27	121.2 (8)
C4—C3—H3	120.3	C29—C28—H28	119.4
C3—C4—C5	119.5 (8)	C27—C28—H28	119.4
C3—C4—H4	120.2	C28—C29—C24	119.8 (7)
C5—C4—H4	120.2	C28—C29—H29	120.1
N1—C5—C4	121.4 (7)	C24—C29—H29	120.1
N1—C5—C6	116.5 (6)	C35—C30—C31	117.6 (6)
C4—C5—C6	122.1 (7)	C35—C30—P2	120.7 (5)
N2—C6—C7	121.2 (7)	C31—C30—P2	121.5 (5)
N2—C6—C5	115.7 (5)	C32—C31—C30	121.1 (6)
C7—C6—C5	123.1 (7)	C32—C31—H31	119.5
C8—C7—C6	118.8 (8)	C30—C31—H31	119.5
C8—C7—H7	120.6	C31—C32—C33	120.9 (7)
C6—C7—H7	120.6	C31—C32—H32	119.6
C7—C8—C9	120.5 (7)	C33—C32—H32	119.6
C7—C8—H8	119.8	C34—C33—C32	118.1 (7)
C9—C8—H8	119.8	C34—C33—H33	121.0
C8—C9—C10	118.1 (8)	C32—C33—H33	121.0
C8—C9—H9	121.0	C33—C34—C35	121.8 (7)
C10—C9—H9	121.0	C33—C34—H34	119.1
N2—C10—C9	123.2 (7)	C35—C34—H34	119.1
N2—C10—H10	118.4	C34—C35—C30	120.6 (7)
C9—C10—H10	118.4	C34—C35—H35	119.7
P1—C11—P2^i^	116.7 (3)	C30—C35—H35	119.7
P1—C11—H11A	108.1		
			
N2—Cu1—N1—C1	−177.1 (6)	Cu1—P1—C11—P2^i^	83.5 (4)
P2—Cu1—N1—C1	−59.2 (6)	C18—P1—C12—C17	48.0 (6)
P1—Cu1—N1—C1	85.8 (6)	C11—P1—C12—C17	156.0 (5)
N2—Cu1—N1—C5	2.0 (5)	Cu1—P1—C12—C17	−75.2 (5)
P2—Cu1—N1—C5	119.9 (4)	C18—P1—C12—C13	−138.8 (5)
P1—Cu1—N1—C5	−95.1 (5)	C11—P1—C12—C13	−30.8 (6)
N1—Cu1—N2—C6	−5.9 (4)	Cu1—P1—C12—C13	98.0 (5)
P2—Cu1—N2—C6	−106.4 (4)	C17—C12—C13—C14	−0.9 (10)
P1—Cu1—N2—C6	93.6 (4)	P1—C12—C13—C14	−174.2 (6)
N1—Cu1—N2—C10	−175.2 (6)	C12—C13—C14—C15	0.9 (12)
P2—Cu1—N2—C10	84.3 (6)	C13—C14—C15—C16	1.3 (13)
P1—Cu1—N2—C10	−75.7 (6)	C14—C15—C16—C17	−3.5 (12)
N1—Cu1—P1—C18	−69.1 (3)	C13—C12—C17—C16	−1.3 (10)
N2—Cu1—P1—C18	−149.1 (3)	P1—C12—C17—C16	172.1 (5)
P2—Cu1—P1—C18	56.7 (2)	C15—C16—C17—C12	3.5 (11)
N1—Cu1—P1—C11	159.5 (3)	C11—P1—C18—C19	108.9 (5)
N2—Cu1—P1—C11	79.5 (3)	C12—P1—C18—C19	−146.6 (5)
P2—Cu1—P1—C11	−74.6 (2)	Cu1—P1—C18—C19	−30.4 (6)
N1—Cu1—P1—C12	45.4 (3)	C11—P1—C18—C23	−66.6 (6)
N2—Cu1—P1—C12	−34.6 (3)	C12—P1—C18—C23	38.0 (6)
P2—Cu1—P1—C12	171.2 (2)	Cu1—P1—C18—C23	154.1 (5)
N1—Cu1—P2—C30	21.0 (3)	C23—C18—C19—C20	1.0 (10)
N2—Cu1—P2—C30	106.0 (3)	P1—C18—C19—C20	−174.6 (5)
P1—Cu1—P2—C30	−103.7 (2)	C18—C19—C20—C21	−2.5 (11)
N1—Cu1—P2—C24	−93.3 (3)	C19—C20—C21—C22	1.9 (13)
N2—Cu1—P2—C24	−8.3 (3)	C20—C21—C22—C23	0.3 (13)
P1—Cu1—P2—C24	142.1 (2)	C21—C22—C23—C18	−1.9 (11)
N1—Cu1—P2—C11^i^	137.2 (3)	C19—C18—C23—C22	1.2 (10)
N2—Cu1—P2—C11^i^	−137.8 (3)	P1—C18—C23—C22	176.7 (5)
P1—Cu1—P2—C11^i^	12.5 (3)	C30—P2—C24—C25	−129.9 (5)
C5—N1—C1—C2	−1.4 (11)	C11^i^—P2—C24—C25	126.0 (5)
Cu1—N1—C1—C2	177.7 (6)	Cu1—P2—C24—C25	−12.4 (6)
N1—C1—C2—C3	0.0 (13)	C30—P2—C24—C29	49.5 (6)
C1—C2—C3—C4	0.8 (14)	C11^i^—P2—C24—C29	−54.6 (6)
C2—C3—C4—C5	−0.1 (15)	Cu1—P2—C24—C29	167.1 (5)
C1—N1—C5—C4	2.0 (10)	C29—C24—C25—C26	0.1 (10)
Cu1—N1—C5—C4	−177.2 (6)	P2—C24—C25—C26	179.5 (5)
C1—N1—C5—C6	−179.1 (6)	C24—C25—C26—C27	−1.8 (10)
Cu1—N1—C5—C6	1.7 (8)	C25—C26—C27—C28	1.5 (11)
C3—C4—C5—N1	−1.3 (13)	C26—C27—C28—C29	0.5 (12)
C3—C4—C5—C6	179.9 (8)	C27—C28—C29—C24	−2.2 (12)
C10—N2—C6—C7	0.3 (10)	C25—C24—C29—C28	1.9 (10)
Cu1—N2—C6—C7	−169.9 (6)	P2—C24—C29—C28	−177.6 (6)
C10—N2—C6—C5	178.7 (6)	C24—P2—C30—C35	32.0 (6)
Cu1—N2—C6—C5	8.5 (7)	C11^i^—P2—C30—C35	139.2 (6)
N1—C5—C6—N2	−6.9 (9)	Cu1—P2—C30—C35	−90.5 (6)
C4—C5—C6—N2	172.0 (7)	C24—P2—C30—C31	−154.5 (5)
N1—C5—C6—C7	171.4 (7)	C11^i^—P2—C30—C31	−47.3 (6)
C4—C5—C6—C7	−9.7 (11)	Cu1—P2—C30—C31	83.1 (5)
N2—C6—C7—C8	−0.2 (12)	C35—C30—C31—C32	−0.5 (9)
C5—C6—C7—C8	−178.5 (8)	P2—C30—C31—C32	−174.2 (5)
C6—C7—C8—C9	0.0 (14)	C30—C31—C32—C33	0.4 (11)
C7—C8—C9—C10	0.2 (14)	C31—C32—C33—C34	0.5 (12)
C6—N2—C10—C9	−0.1 (11)	C32—C33—C34—C35	−1.2 (12)
Cu1—N2—C10—C9	168.8 (6)	C33—C34—C35—C30	1.1 (12)
C8—C9—C10—N2	−0.1 (13)	C31—C30—C35—C34	−0.2 (10)
C18—P1—C11—P2^i^	−52.5 (4)	P2—C30—C35—C34	173.6 (6)
C12—P1—C11—P2^i^	−159.4 (3)		

Symmetry codes: (i) −*x*+1, −*y*+1, −*z*+1.

## References

[bb1] Armaroli, N. (2001). *Chem. Soc. Rev.* **30**, 113–124.

[bb2] Bruker (1997). *SAINT* and *SMART* Bruker AXS Inc., Madison, Wisconsin, USA.

[bb3] Diez, J., Gamasa, M. P. & Gimeno, J. (1987). *J. Chem. Soc. Dalton Trans.* pp. 1275–1278.

[bb4] Ho, D. M. & Bau, R. (1983). *Inorg. Chem.* **22**, 4073–4079.

[bb5] Jia, W. L., McCormick, T., Tao, Y., Lu, J. P. & Wang, S. N. (2005). *Inorg. Chem.* **44**, 5706–5712. 10.1021/ic0504893 16060621

[bb6] Jin, S. W., Wang, D. Q., Wang, X. L., Guo, M. & Zhao, Q. J. (2008). *J. Inorg. Organomet. Polym.* **18**, 300–303.

[bb7] Kuang, S. M., Cuttell, D. G., McMillin, D. R., Fanwick, P. E. & Walton, R. A. (2002). *Inorg. Chem.* **41**, 3313–3322. 10.1021/ic0201809 12055011

[bb8] Sheldrick, G. M. (1996). *SADABS* University of Göttingen, Germany.

[bb9] Sheldrick, G. M. (2008). *Acta Cryst.* A**64**, 112–122. 10.1107/S0108767307043930 18156677

[bb10] Stockland, R. A., Janka, M., Hoel, G. R., Rath, N. P. & Anderson, G. K. (2001). *Organometallics*, **20**, 5212–5219.

[bb11] Yam, V. W. W., Fung, W. K. M. & Cheung, K. K. (1997). *Chem. Commun.* pp. 963–964.

